# Does synbiotic supplementation affect body weight, body mass index, and high-sensitivity C-reactive protein levels in patients with type 2 diabetes? Protocol for a systematic review and meta-analysis

**DOI:** 10.1097/MD.0000000000018197

**Published:** 2019-12-10

**Authors:** Yuan Chen, Zhenhua Li, Maoyi Yang, Jiacheng Shui, Rensong Yue

**Affiliations:** a Hospital of Chengdu University of Traditional Chinese Medicine; b Chengdu University of Traditional Chinese Medicine, Chengdu, Sichuan; c Department of Obstetrics and Gynecology, Qufu Hospital of Traditional Chinese Medicine, Qufu, Shandong, China.

**Keywords:** body mass index, body weight, high-sensitivity C-reactive protein, protocol, synbiotic, type 2 diabetes mellitus

## Abstract

**Background:**

The number of patients with type 2 diabetes mellitus (T2DM) is surging currently. Synbiotic as a supplement based on gut microbiota may be beneficial to improve the metabolism of T2DM. However, the results of clinical studies show that the role of synbiotic in weight management in patients with T2DM is controversial. In this context, we have formulated this protocol. The study will evaluate the effects of synbiotic supplementation on body weight, body mass index (BMI), and high-sensitivity C-reactive protein (hs-CRP) levels in patients with T2DM.

**Methods:**

The electronic databases PubMed, Embase, and the Cochrane Library will be searched for relevant literature from inception. Literature search, data extraction, and methodological quality assessment will be carried out independently by two researchers. All randomized controlled trials (RCTs) that met the criteria will be included. A meta-analysis will be conducted using weighted mean difference (WMD) and 95% confidence interval (CI) as effect measures.

**Results:**

This systematic review and meta-analysis will mainly assess the effects of synbiotic supplementation on body weight and BMI in T2DM patients. Secondary outcome indicators will include hs-CRP.

**Conclusion:**

This systematic review and meta-analysis will quantify the value of synbiotic supplement in weight management of patients with T2DM through a comprehensive evaluation of the current clinical evidence, so as to provide a basis for clinical application.

**PROSPERO registration number:**

CRD42019132974.

## 1 Introduction

The incidence of diabetes mellitus has risen sharply and its prevalence poses a major threat to global health. According to the 8th edition of the International Diabetes Federation Diabetes Atlas, about 425 million people between the ages of 20 and 79 years worldwide live with diabetes as of 2017. This number is still growing and has been predicted to reach 629 million by 2045.^[1]^ Among this large population of patients with diabetes, type 2 diabetes mellitus (T2DM) accounts for more than 90% cases,^[2]^ and is understandably the focus of global diabetes prevention and treatment.

Although multiple factors such as genetics and environment are important pathogenic drivers for T2DM, studies have shown that obesity and poor lifestyle are mainly responsible for the high global incidence of T2DM.^[3]^ A previous survey found that the incidence of T2DM and obesity showed parallel rising trends,^[4]^ with about 80% patients with T2DM being overweight. Thus, overweight and obesity deserve more attention than other factors given their clear association with T2DM.^[5,6]^ There is evidence that weight loss of more than 15 kg may reverse T2DM status.^[7]^ Therefore, effective control of weight gain is beneficial for patients with T2DM. Weight control requires a combination of multiple approaches such as diet, exercise, nutrition, medication, and, in more extreme cases, surgery. Although lifestyle interventions are key to weight loss, lifestyle modifications alone are inadequate and often ineffective for achieving adequate weight loss. This may be an even greater challenge for patients with T2DM in particular, as some treatment options are associated with weight gain.^[8,9]^

The gut microbiome is known as the “second genome” of humans^[10]^ and is closely related to host health. A recent study has further clarified the causal relationship between gut microbiota and metabolic diseases, such as obesity and T2DM.^[11]^ Other studies^[12,13]^ have shown that the structure and richness of fecal bacteria are related to basic metabolic markers of the body such as glucose and lipid levels, body weight, and inflammation status. The gut microbiota and its metabolites such as short-chain fatty acids (SCFAs) can affect host-energy metabolism by regulating food intake, energy harvesting, and consumption, which is of great significance for weight control. Interestingly, the gut microbiota exhibits plasticity and responds to the host's lifestyle.^[14]^ Therefore, gut microbiota intervention may provide a new therapeutic approach for weight management in patients with T2DM. In this context, supplementation with probiotics or synbiotics as a method to regulate the gut microecosystem has attracted the attention of many researchers.

Probiotics and prebiotics are both health-beneficial substances, and when combined into synbiotics, they can produce synergistic effects to provide comprehensive therapeutic benefits. Some studies have shown that supplementation with synbiotics can improve glucose and lipid metabolism in patients with diabetes^[15]^; however, the effects of synbiotics on indicators of body weight and inflammation are still controversial. Moreover, to the best of our knowledge, no meta-analysis has been conducted to evaluate the independent effects of synbiotic supplementation in patients with T2DM in regard to body weight and inflammation. Accordingly, we will conduct a systematic review and meta-analysis to evaluate the effects of synbiotic supplementation on body weight, BMI, and hs-CRP levels in patients with T2DM. This review will effectively summarize the existing evidence to provide a basis for clinical decision-making.

## 2 Methods

### 2.1 Study registration

This systematic review and meta-analysis protocol were reported according to the Preferred Reporting Items for Systematic Reviews and Meta-analysis Protocols (PRISMA-P) checklist^[16]^ and has been registered on the PROSPERO (registration no. CRD42019132974).

### 2.2 Data sources and search strategies

To identify relevant studies, an online search of the of PubMed, Embase, and Cochrane Library (CENTRAL) databases will be conducted from inception. Two researchers (Maoyi Yang and Jiacheng Shui) will carry out the searches independently. The literature search will be restricted to studies published in English. Medical Subject Headings (MeSH) terms and their synonyms were used to maximize the sensitivity of the search. The specific search strategies were as follows: (“synbiotics [MeSH]” OR “synbiotic” OR “symbiotic” OR “synbiotic agent”) combined with (“Diabetes Mellitus, Type 2[MeSH]” OR “Type 2 Diabetes Mellitus” OR “Type 2 Diabetes” OR “T2DM” OR “Diabetes Mellitus” OR “Diabetes” OR “Diabetic” OR “DM” OR “Hyperglycemia”). The search strategy for PubMed was shown in Table 1. Further, the reference lists of all identified articles will be scrutinized to identify eligible studies not found through the original database searches.

**Table 1:**
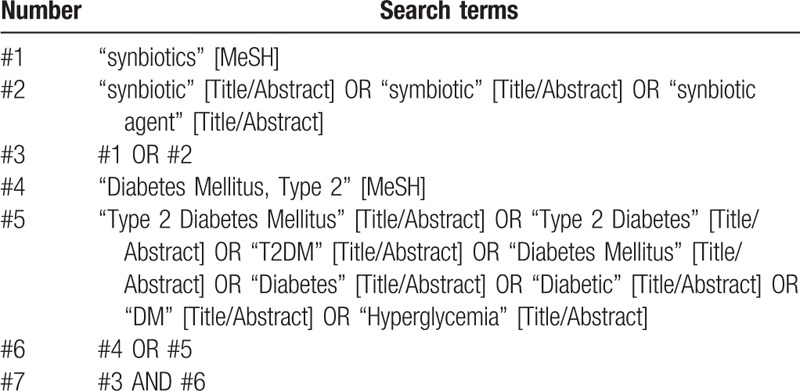
Search strategy for PubMed.

### 2.3 Study selection

**Figure 1:**
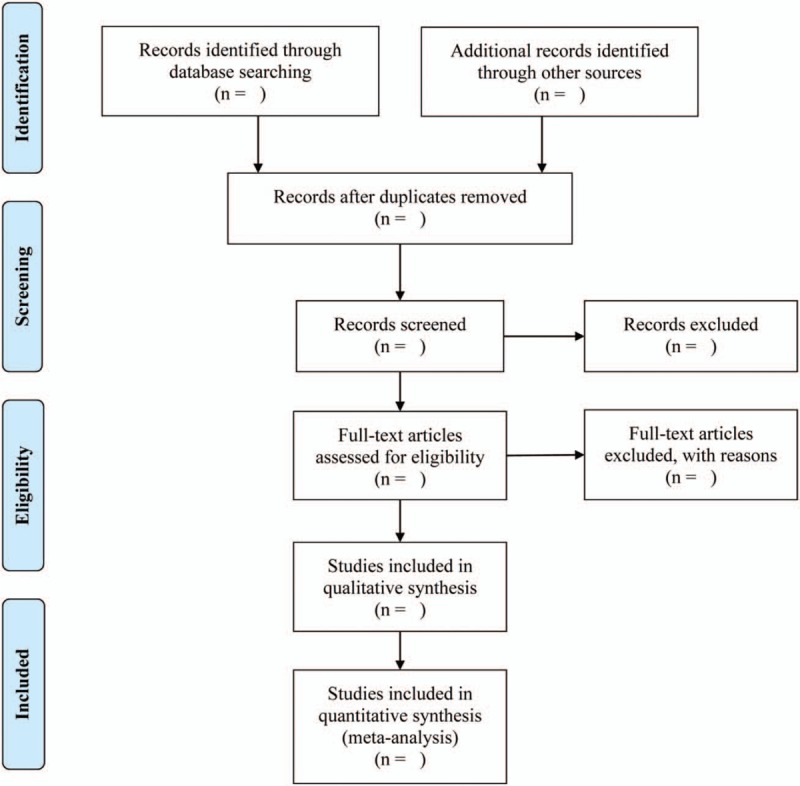
Flowchart of the methods used to search and select literature.

After elimination of duplicate literature, the remaining studies will be used for further screening. Each study will be independently evaluated by 2 authors (Maoyi Yang and Jiacheng Shui) based on the title and abstract to screen out potentially eligible articles. Then, the authors will conduct a full-text evaluation of potentially eligible articles. Endnote X8 (Thomson Reuters, USA) will be used to manage our literature. The process of retrieving and filtering articles is shown in Figure 1.

#### 2.3.1 Study type

All randomized controlled trials (RCTs) of supplementary synbiotics conducted in T2DM patients will be included.

#### 2.3.2 Study object

Trials will be included if they involved adult patients diagnosed T2DM (age, >18 years).

#### 2.3.3 Interventions

Synbiotics were the only supplement used in the intervention group, and placebo was used in the control group.

#### 2.3.4 Outcomes

The included studies must contain at least 1 of following outcomes of interest for this review: body weight, BMI, or hs-CRP; and data for these indicators, including the baseline and endpoint values or net changes between them with mean and standard deviation (SD) were available.

#### 2.3.5 Exclusion criteria

Studies will be excluded if they:

(1) did not state sufficient information on the above-mentioned outcome indicators;
(2) were abstracts, case reports, study protocols, review articles, letters, or did not feature a full-body of text; or
(3) reported duplicate data.

### 2.4 Data extraction

Two reviewers, Maoyi Yang and Jiacheng Shui, will independently extract the data from all candidate studies using a standardized data record form. Discrepancies will be resolved by consensus or by referring to a third author for adjudication (Yuan Chen). The following data will be collected from the included studies:

(1) trial characteristics including first author, publication year, study design and sample size(n), therapy information (synbiotics source and dose), and duration of intervention (weeks);
(2) participants’ information including country and patient age (years); and
(3) outcomes of interest—body weight, BMI, and hs-CRP levels.

### 2.5 Risk of bias assessment

The methodological quality of all included studies will be assessed according to the Cochrane Collaboration risk of bias tool.^[17]^ The following seven items will be used to assess the risk of bias: random sequence generation (selection bias), allocation concealment (selection bias), blinding of participants and personnel (performance bias), blinding of outcome assessment (detection bias), incomplete outcome data (attrition bias), selective reporting (reporting bias), and other bias. The risk of bias in each item was divided into low risk, high risk, or unclear risk. The assessment will be performed independently by 2 authors (Yuan Chen and Zhenhua Li). Disagreements will be resolved through consensus.

### 2.6 Data analysis

#### 2.6.1 Data synthesis and statistical analysis

The outcomes of net change in body weight, BMI, and hs-CRP from baseline will be used to calculate the weighted mean differences (WMD) and 95% confidence intervals (CIs) between the intervention and control groups. The SD of the mean change value will be calculated according to the formula if it was not reported. The correlation coefficient will be estimated based on the reported baseline and end-point values and changes in body weight, BMI and hs-CRP in the included studies. Cochran *Q* test will be used to evaluate inter-study heterogeneity. The magnitude of heterogeneity will be measured by the Higgins I-squared (*I*^*2*^) statistic. In each analysis, *I*^*2*^ values of 25%, 50%, and 75% defined low, moderate, and high heterogeneity, respectively.^[18]^*P* value <.1 and *I*^*2*^ >50% indicated significant heterogeneity. The data will be then pooled from a random-effects model; otherwise, a fixed-effects model will be used. Overall effects will be assessed using Z-scores, and *P* values <.05 will be considered statistically significant. If quantitative synthesis is not appropriate, we will only objectively describe the trial results. All statistical analyses will be performed using RevMan (Version 5.3, Cochrane, Oxford, UK) and STATA (Version 12.0, College Station, TX) software.

#### 2.6.2 Subgroup analyses

To identify the potential sources of inter-study heterogeneity, a pre-planned subgroup analysis will be performed based on the number of probiotics contained in synbiotics, the intervention duration of the study, and the form of synbiotic supplementation.

#### 2.6.3 Sensitivity analysis

Sensitivity analysis will be performed to evaluate the robustness of the pooled result.

#### 2.6.4 Assessment of publication bias

If more than 10 trials are included in the meta-analysis, publication bias will be assessed using funnel plots and the Egger test; *P* value <.1 will be considered to indicate statistically significant publication bias.

#### 2.6.5 Grading the quality of evidence

In this study, the tool of GRADE system will be used to evaluate the quality of evidence. Five items, namely, risk of bias, publication bias, indirectness, imprecision, inconsistency, will be evaluated. The quality of evidence will be divided into 4 levels: high, moderate, low, and very low.

#### 2.6.6 Patient and public involvement

Patient and public were not involved in this study.

#### 2.6.7 Ethics and dissemination

This study does not require ethical approval, and the results of systematic review and meta-analysis will be published in a peer-reviewed journal.

## 3 Discussion

The current global epidemic trend of T2DM is closely related to overweight or obese status. Studies have shown that the incidence of diabetes increases with the increase of BMI^[19]^; for patients with T2DM, being overweight/obese makes glycemic control difficult and exacerbates the risk of diabetes-related complications, which in turn increases treatment costs.^[20]^ Therefore, weight management is very important in the prevention and treatment of T2DM. Imbalance of energy metabolism is considered an important cause of being overweight/obese, and several studies have shown that gut microbiota play an important role in regulating energy balance.^[21,22]^ The association between gut microbiota and overweight/obese status has prompted researchers to explore the effects of interventions targeting gut microbiota on weight. Probiotics and prebiotics are considered nutritional agents that improve host health by modifying the gut microbiota. Understandably, synbiotics—a synergistic combination of the 2—are attracting increasing attention. Tabrizi et al^[15]^ found that synbiotic supplementation could improve blood lipid levels in patients with diabetes; Hadi et al^[23]^ studied the effect of synbiotic supplementation in obese patients, and found that synbiotics might have a modest effect on body weight (WMD: –0.80 kg) and waist circumference (WMD: -2.07 cm), but no significant effect on BMI and body fat. At present, similar clinical trials have been carried out in patients with T2DM, but the results are still controversial, so the meta-analysis of the current clinical evidence will be meaningful.

Systemic low-grade inflammation is a negative consequence of obesity, as this inflammatory state can lead to insulin resistance, energy imbalance, and subsequently T2DM induction. Inflammation establishes a strong link between obesity and T2DM,^[24]^ and T2DM is now considered a chronic inflammatory disease. Based on this concept, anti-inflammatory therapy may have potential benefits in the management of T2DM. The anti-inflammatory effects of synbiotics in chronic intestinal diseases have been demonstrated.^[25]^ Therefore, it is necessary to further evaluate the effect of synbiotic supplement on inflammatory indexes in patients with T2DM.

This systematic review and meta-analysis will quantify the value of synbiotic supplement in weight management of patients with T2DM, so as to provide available evidence for clinical application. we will conduct a comprehensive literature search and evaluation report in strict compliance with the PRISMA standard to minimize bias in the implementation process.

## Author contributions

**Conceptualization:** Yuan Chen, Zhenhua Li, Rensong Yue.

**Data curation:** Maoyi Yang, Jiacheng Shui.

**Formal analysis:** Yuan Chen, Zhenhua Li, Maoyi Yang.

**Investigation:** Yuan Chen, Maoyi Yang, Jiacheng Shui.

**Methodology:** Yuan Chen, Zhenhua Li, Rensong Yue.

**Project administration:** Rensong Yue.

**Software:** Yuan Chen, Maoyi Yang, Jiacheng Shui.

**Supervision:** Yuan Chen, Rensong Yue.

**Visualization:** Yuan Chen, Zhenhua Li.

**Writing – original draft:** Yuan Chen

**Writing – review & editing:** Yuan Chen, Zhenhua Li, Rensong Yue, Maoyi Yang, Jiacheng Shui.
